# Smart Nanotheranostics Responsive to Pathological Stimuli

**DOI:** 10.3389/fbioe.2020.00503

**Published:** 2020-05-25

**Authors:** Alessandro Parodi, Magdalena Rudzinska, Stefano Leporatti, Yuri Anissimov, Andrey A. Zamyatnin

**Affiliations:** ^1^Institute of Molecular Medicine, Sechenov First Moscow State Medical University, Moscow, Russia; ^2^CNR NANOTEC - Istituto di Nanotecnologia, Polo di Nanotecnologia, Lecce, Italy; ^3^School of Environment and Sciences, Griffith University, Gold Coast, QLD, Australia; ^4^Belozersky Institute of Physico-Chemical Biology, Lomonosov Moscow State University, Moscow, Russia

**Keywords:** nanotheranostics, smart nanoparticles, pH-responsive theranostics, ROS-responsive theranostics, Enzyme-responsive theranostics

## Abstract

The development of nanotheranostics represents one of the most dynamic technological frontiers in the treatment of different pathological conditions. With the goal in mind to generate nanocarriers with both therapeutic and diagnostic properties, current research aims at implementing these technologies with multiple functions, including targeting, multimodal imaging, and synergistic therapies. The working mechanism of some nanotheranostics relies on physical, chemical, and biological triggers allowing for the activation of the therapeutic and/or the diagnostic properties only at the diseased site. In this review, we explored new advances in the development of smart nanotheranostics responsive to pathological stimuli, including altered pH, oxidative stress, enzymatic expression, and reactive biological molecules with a deep focus on the material used in the field to generate the particles in the context of the analyzed disease.

## Introduction

Nanotheranostic development perhaps represents the highest level of technological advance in the nanomedicine field, and it aspires to combine in the same delivery platform therapeutic and diagnostic properties (Wong et al., 2020). Nanotheranostics are nanoparticles designed to provide real-time information about drug biodistribution, release, and targeted treatment *in vivo*, representing one of the last frontiers in personalized medicine (Jo et al., 2016). Nanotheranostics are usually generated through complex synthetic protocols (Silva et al., 2019) necessary to transfer multiple functions to the same delivery platform. For this reason, in several cases, nanotheranostics' targeting simply relies on particle passive accumulation in the diseased tissue *via* enhanced permeability and retention effect (EPR) usually achieved with biological coating (i.e., albumin, peptides) or polyethylene glycol (PEG) surface modification.

However, beyond exploiting a specific targeting, nanotheranostic selectivity for the pathological area can derive from a specific “responsiveness” of the carriers to an external stimulus (Sneider et al., 2017). This “activation trigger” (e.g., near-infrared light) is usually remotely applied directly in the area of interest (Wang et al., 2017b) and has benefits of non-invasiveness. Typical diagnostic or therapeutic carriers are generally in the “on” state (Zhao et al., 2017), and their detection and payload release occur from the moment of their administration. On the other hand, the designing of carriers with “off-on” theranostic properties (Yu et al., 2018) can favor a personalized assessment of the amount of drug that effectively reaches the pathological site. For this reason, responsive nanotheranostics have the potentialities to open new avenues for optimizing and controlling the treatment dose and repetition (Fan et al., 2016; Yang et al., 2016b). In this scenario, it seems almost impossible to increase further the level of complexity of these technologies. To date, many theranostics can permit multimodal imaging and therapy for enhancing diagnostic accuracy and treatment efficacy (Dong et al., 2016). However, new trends in nanomedicine aim at imparting the carriers with responsiveness to the biological environment conditions. In other words, the physiological alterations that differentiate diseased from healthy tissue can serve as “triggers” to turn *in vivo* the nanotheranostics “on” (Ma et al., 2016). Also, these concepts showed the potential to improve current experimental protocols. For example, nanotheranostics that can enhance their detection signal in response to precise biological stimuli could mitigate the background noise issues related to the imaging of fluorescently modified carriers (Wu et al., 2014).

The working mechanism of the stimuli-responsive carriers, often referred to as “smart” theranostics, usually depends on functional molecules or chemical linkers used to assemble the nanoparticles (Karimi et al., 2016). However, in some cases, it is the intimate structure of the particles that changes or responds to the environmental conditions, like in the case of polymers (Li et al., 2019a). In this review, we explored recent advances in the development of nanotheranostics that can respond to biological stimuli. These carriers were designed to provide their curative and/or diagnostic properties only in the pathological site, exploiting altered chemical and biological features of the diseased tissue. These concepts, as well as the field of nanomedicine, are traditionally applied to cancer disease because tumor tissue undergoes profound changes in cell metabolism, generating significant variations in local pH and oxidative stress. Sometimes the nanoparticles were designed to respond to more than one biological stimuli as well as in combination with physical stimuli that can be remotely administered. Also, tumor growth very often depends on the differential expression of enzymes that can be exploited as biological triggers as well (Xiao et al., 2018). However, recent evidence demonstrated that the concepts of biologically responsive nanotheranostic could be beneficial also for other pathological conditions, expanding the application of these technologies to a new whole portfolio of clinical conditions.

## pH-Responsive Theranostics

In the area of cancer therapy, pH represents an essential cue of differentiation compared to healthy tissue. Even though not all the regions of the tumor become significantly acidified, changes in cancer metabolism [i.e., the Warburg effect (Tekade and Sun, 2017; Shamsi et al., 2018)], can induce an overall average decrease of 0.2–0.4 points, although values <6 were registered as well (Liu et al., 2014a). In this scenario, the tumor microenvironment pH can eventually represent a targetable characteristic. Also, after cell internalization, the nanoparticles are usually sequestered in the endolysosomal compartment, where the pH drops significantly below 5 (Wang et al., 2017a), making these organelles optimal targets for pH-responsive technologies.

pH-responsive properties can be coupled with the activation of different therapeutic mechanisms, including reactive oxygen species (ROS)-based cytostatic therapeutics, as well as different imaging modalities. In this context, sono-, photo-, and chemodynamic therapies are treatments in which the cytostatic properties rely on the overproduction of ROS. In sono- and photodynamic therapy (PDT), ROS can be generated upon an external stimulus. In contrast, chemodynamic therapy (CDT) depends just on the chemical properties of the carriers or the payload responsible for ROS generation, usually when interacting with the cellular H_2_O_2_ (Lin et al., 2018).

The pH-responsiveness can derive from pH-sensitive linkers used to stabilize the particles (Kanamala et al., 2016) or its payload in the carrier structure. An extensive overview of this topic can be found in Cao et al. (2019). Acotinyl linkers were intensely investigated in the field to impart pH responsiveness to the carriers. For example, Zhu et al. generated pH-responsive theranostics incorporating gold nanoparticles in poly(amidoamine) dendrimers (Zhu et al., 2018). The gold nanoparticles represented an optimal contrast agent in computed tomography (CT) imaging. The dendrimers were modified on their surface with folic acid *via* EDC chemistry to provide the particles with high tumor targeting. Cis-aconitic anhydride pH-sensitive linkers were used to conjugate Doxorubicin (DOX) to the particles. Drug release was triggered by the acidic conditions (typical of the tumor microenvironment and the endosomal compartment) even though *in vivo* proof of the efficacy of this technology was not reported. Similarly, nanotheranostics were generated with bovine serum albumin (BSA) linked to the porphyrin photosensitizer pheophorbide-a *via* cis-aconityl pH-sensitive linkers. Stable nanoparticles were generated by complexing this structure with graphene oxide *via* π-π stacking and hydrophobic interactions (Battogtokh and Ko, 2016). The carriers were further modified with pegylated folate to impart the system with extended circulation properties and tumor targeting. Porphyrins are known for their therapeutic potential (PDT) and fluorescent properties, but their administration requires encapsulation since they are strongly hydrophobic (Yan et al., 2015). This complex facilitated dual therapy through PDT and photothermal therapy (PTT) due to the porphyrin payload and the graphene oxide, respectively, upon irradiation at 670 nm. *In vivo*, compared with the free administered photosensitizer, the particle highly accumulated in the tumor tissue through EPR effect. Here they could release their payload due to the pH-sensitive linkers both in the acidic tumor microenvironment and well as in the cell cytoplasm after internalization favored by the folate functionalization. The pH-sensitive mechanism was fundamental to activate the theranostic properties of the porphyrin since this molecule is affected by aggregation-caused quenching (ACQ) effect when encapsulated.

On the other hand, different materials can be manipulated in the nanoscale and dissolve (releasing a payload) in acidic pH. Calcium carbonate (CaCO_3_) nanoparticles represent a well-investigated delivery platform (Idris et al., 2019; Vidallon et al., 2020) in this field. At physiological pH, CaCO_3_ nanoparticles are stable, but under acid conditions, they degrade into Ca^2+^ and CO_2_, releasing whatever payload was previously loaded. Pegylated CaCO_3_ nanoparticles were synthesized by gas diffusion-reaction in combination with the photodynamic theranostic agent chlorin e6 (Ce6) (Dong et al., 2016). Like porphyrins, Ce6 is *per se* a theranostic because it can serve as a therapeutic tool for PDT while emitting a detectable fluorescent signal upon near-infrared light (NIR) irradiation (Liu et al., 2014b). The particles were also doped with Mn^2+^, to favor the photosensitizer precipitation with the CaCO_3_. Mn^2+^ also allowed for detecting particle degradation and payload release *via* magnetic resonance imaging (MRI), and it provided a mean for CDT increasing ROS levels *via* Fenton-like reaction with tissue H_2_O_2_. In this chemical reaction a transition metal (i.e., Fe, Al, Mn, Cu, Zn) interacting with H_2_O_2_ is oxidized while catalyzing the formation of a hydroxyl radical and a hydroxide ion (Das et al., 2015). The system was characterized by a mesoporous structure compatible with the loading of conventional chemotherapeutics like DOX that, in acidic conditions, was released as well. The particles were also modified with PEG allowing for extravasation in the tumor microenvironment *via* EPR. Under the acidic conditions of cancer tissue, they could exert their theranostic properties demonstrating high cytostatic properties both *in vitro* and *in vivo* against a model of breast cancer, The authors demonstrated that chemotherapy (DOX), CDT (Mn^2+^) and PDT (Ce6) could work synergistically against cancer cell growth (Dong et al., 2016) accomplishing multimodal therapy.

**Figure 1:**
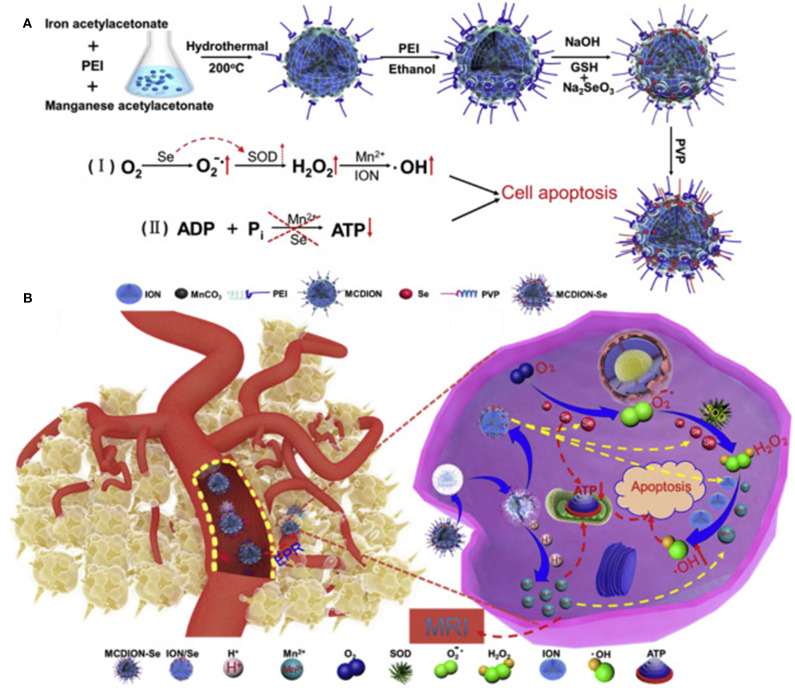
**(A)** MCDION-Se synthesis. Iron (III) acetylacetonate and manganese acetylacetonate were used as precursors for particle synthesis *via* solvent, thermal decomposition with polyethyleneimine (PEI) as a surfactant. PEI also allowed for further modification with Se nanoparticles *via* electrostatic interactions between the positively charged polymer and the negatively charged Se. Polyvinylpyrrolidone (PVP) was used to stabilize the system. The scheme also illustrates the synergistic action of Mn and Se in inducing the formation of ROS and consequent cell apoptosis. In particular, the selenium nanoparticles induced the formation of superoxide radicals and the activation of the enzyme superoxide dismutase (SOD) to generate H_2_O_2._ Manganese and iron oxide nanoparticles catalyzed further conversion of H_2_O_2_ to hydroxyl radical. Mn and Se also negatively impacted on ATP synthesis. **(B)** After IV administration, the particles extravasated in the tumor microenvironment *via* EPR. Here they can be internalized by cancer cells and induce a cascade of reactions that increase cell apoptosis *via* ROS production and inhibition of ATP synthesis. Reproduced with permission from Xiao et al. (2019).

Another example of pH-responsive structure was proposed by Xiao et al. (2019) that developed a theranostic platform named MCDION-Se able to provide at the same time chemodynamic and limotherapy while representing an optimal contrast agent for MRI application. The system was composed of a core of manganese carbonate-deposited iron oxide nanoparticles coated with negatively charged selenium nanoparticles coordinated to the core *via* polyethylenimine (PEI) (Figure 1). The manganese carbonate in the system could easily dissolve in slightly acidic conditions releasing Mn^2+^ for CDT and MRI while inhibiting ATP generation. On the other hand, the presence of the selenium enhanced the formation of H_2_O_2_, fueling the Fenton-like reaction catalyzed by the iron oxide nanoparticles to form ROS that further inhibited ATP synthesis. The combination of these elements was designed to breakdown in the tumor microenvironment and tumor cells after internalization, triggering different cascades of events affecting the energetic metabolism (limotherapy) while accelerating cell apoptosis *via* CDT. *In vivo*, the particles showed a higher circulation time and tumor accumulation than free Mn^2+^, probably *via* the EPR effect even though specific modifications to favor this phenomenon were not described. Manganese was also exploited by Liu et al. that designed PEG-coated MnO_2_ nanoparticles stabilized with BSA. The particles were loaded with the radiosensitizer hafnium and a prodrug form of cisplatin. The core of MnO_2_ could catalyze the conversion of tumor H_2_O_2_ to O_2_ to revert hypoxic tumor conditions while the hafnium synergistically increased radiotherapy efficiency. After particle extravasation *via* EPR, the particles dissolved in acidic conditions offering an optimal contrast agent for MRI. Besides, the prodrug could be internalized by tumor cells and converted to cisplatin *via* cellular glutathione. The system provided a dual therapy mechanism (chemo and radiotherapy) effective against a model of breast cancer *in vivo* (Liu et al., 2017).

Finally, it is worth reporting the work of Li et al. (2020a) that exploited the acidic properties of the tumor microenvironment to increase the generation of ROS in hypoxic tissue, where this strategy is usually not achievable due to the lack of oxygen (Chen et al., 2015). The system was composed of biodegradable magnetic mesoporous nanocubes that efficiently induced hyperthermia when exposed to an external high frequency alternating magnetic field. The particles were loaded with Vitamin C to selectively kill cancer cells through the formation of the ascorbate radical and H_2_O_2_ (Yun et al., 2015; Lv et al., 2018). Its release was induced by the material phase-change that turned from the solid to the liquid state when the surrounding temperature was higher than 38°C. More importantly, in acidic conditions, the metallic nature of these nanoparticles induced the transformation of H_2_O_2_ into O_2_ as well as its further conversion to hydroxyl radical *via* Fenton reaction (Cao et al., 2018) while serving as an optimal T_2_ MRI contrast agent. The system showed theranostic properties both *in vitro* and *in vivo*, where the particle biodistribution could be tracked *via* MRI. Upon application of the external magnetic field, the generated hyperthermia induced the release of vitamin C with its consequent tumor-killing properties.

As shown in this section, pH responsiveness can be imparted through different chemical linkers and materials sensitive to pH to trigger the therapeutic and/or the imaging properties. The responsiveness of most of these technologies depends on the sensitivity of their ultrastructure to the pH. More importantly, the pH-responsiveness could be exploited for increasing tumor oxygen levels for improving radiotherapy effectiveness or CDT in hypoxic tumor conditions. Finally, pH-sensitivity can be coupled with other mechanisms of responsiveness that rely on external stimuli to increase the carrier therapeutic and diagnostic properties.

## ROS-Responsive Theranostics

Due to their high metabolism and accelerated growth, cancer cells are characterized by an increased generation of ROS (Trachootham et al., 2009). On the other hand, this enhanced oxidative stress is compensated by a higher average content of glutathione (Desideri et al., 2019) that in the field of nanotheranostics is exploited as a biological trigger as well, and will be discussed in the last section of this review. It is worth mentioning that all ROS-responsive mechanisms eventually depend on a biological trigger because their working mechanism depends on the oxygen content in the tissue, and they are ineffective in hypoxic regions (Chen et al., 2015). In some cases, the ROS generation is catalyzed directly by the nanoparticles, that due to the properties of their synthesis material, can activate other features of the carriers like payload release (Sun et al., 2018).

In the field of PDT, new evidence are indicating that the best treatment efficacy occurs when the photosensitizer is coupled with a chemotherapeutic agent generating a synergistic effect. To achieve this goal, it is mandatory designing particles in which the ROS can induce a burst release of the therapeutic (Yang et al., 2016a; Zhou et al., 2017). For this reason, the carriers needed to generate ROS as well as being sensitive to these chemical species. To this goal, Sun et al. (2018) developed nanoparticles of pegylated polyphosphate crosslinked with a thioketal linker *via* (A2 + B3) type polycondensation. The carriers were loaded with the photosensitizer Ce6 and DOX. Under NIR light irradiation, the photosensitizer induced the generation of ROS, while favoring the degradation of the thioketal linker resulting in a burst release of DOX. This strategy was effective in overcoming DOX drug resistance i*n vitro* in a cell line of breast cancer (MCF-7/ADR) overexpressing P-glycoprotein. *In vivo*, the particle demonstrated good tumor accumulation *via* the EPR effect as well as high tumor-killing properties. The system showed bimodal imaging capabilities achieved both *in vitro* and *in vivo*, exploiting Ce6 photoacoustic (PA) properties and gadolinium (loaded in the particles *via* its natural affinity for Ce6) that allowed for efficient MRI. Supported by dual-modal imaging, the tumor sites could be precisely irradiated, sparing healthy organs and reducing kidney and liver toxicity.

Within the cell cytoplasm, mitochondria are the most active organelles in terms of ROS generation (Dunn et al., 2015) and, compared to healthy cells, cancer cells showed a higher mitochondrial membrane potential favoring their targeting *via* internalized cationic molecules (Modica-Napolitano and Aprille, 2001). Inspired by this evidence, Yue et al. developed a nanotheranostic composed of triphenylphosphine, a positively charged molecule with a high affinity for the mitochondria, condensed *via* amphiphilic block polymerization with camptothecin (CPT) and the photosensitizer zinc phthalocyanine (ZnPC) for PDT generation. The drug conjugation occurred through pegylated thioketal linkers sensitive to ROS (Yue et al., 2016). The particles were designed to extravasate in the tumor microenvironment *via* EPR, while tumor cell internalization and mitochondrial targeting were achieved through the membrane penetrating properties of the positively charged triphenylphosphine. The ROS generated by the mitochondria and *via* PDT favored the release of the CPT (topoisomerase-inhibitor) and a dual therapy mode. The system could be detected *via* fluorescence imaging *in vitro* and *in vivo* (when ZnPC was replaced by Ce6) in a subcutaneous model of lung cancer. However, for a more comprehensive description of remotely responsive activated technologies, we suggest the readers look elsewhere (Kim et al., 2013; Zhang et al., 2016) since this review focuses mostly on the nanoplatforms that are activated by tissue- or cell-generated ROS.

**Figure 2:**
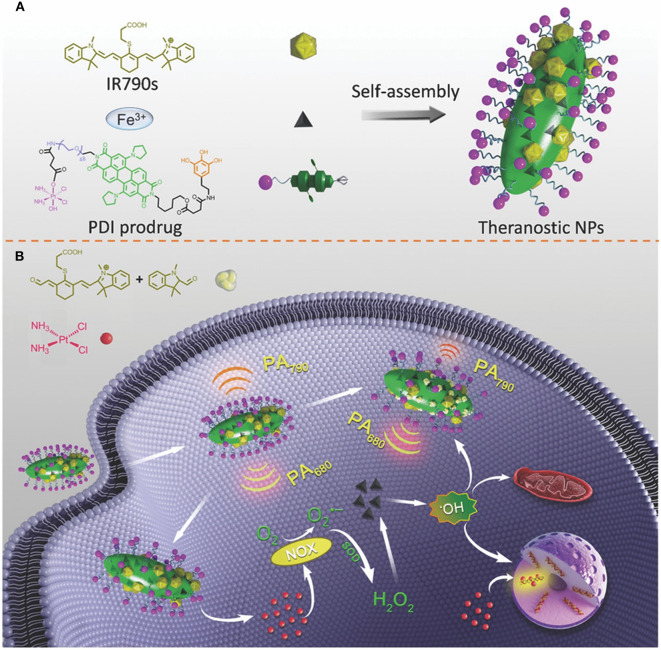
**(A)** Self-assembly of perylene diimide cisplatin prodrug and the infrared dye IR790 in the presence of ferric ions. One of the amide groups of the PDI was modified with polyphenols coordinating the ferric ions necessary for catalyzing H_2_O_2_ in hydroxyl radical in acidic conditions. The second amide of the PDI was conjugated with PEG that increased nanoparticle solubility and allowed for further modification with the cisplatin prodrug. **(B)** The working mechanism of the system. After cancer cell internalization, the cisplatin induced the activation of the nicotinamide adenine dinucleotide phosphate oxidase (NOX) transforming molecular oxygen in O^.−^ with consequent generation of H_2_O_2_ *via* superoxide dismutase. The hydrogen peroxide is further transformed into hydroxyl radicals by the ferric ions inducing cell apoptosis. ROS formation degraded IR790. The measurement of the perylene diimide/IR790 absorption ratio could be used for ratiometric PA imaging of the ROS formation. Reproduced with permission from Yang et al. (2018b).

In this context, the probe IR790s was recently used to generate a platform named perylene diimide –IR790s–Fe/Pt NPs to detect and trace ROS generation through ratiometric photoacoustic (PA) imaging. Perylene diimide showed a strong NIR light absorption at 680 nm, while IR790 absorbed light at 790 nm. The structure of IR790 could be cleaved by ROS, with a consequent decrease of its adsorption at 790 nm. Ratiometric PA imaging was achieved by irradiating the carriers at 680 and 790 nm. The increase in the Ab_680_/Ab_790_ value was directly proportional to the ROS concentration. ROS generation was facilited by cisplatin conjugated on the surface of the particles *via* PEG and ferric anions chelated by the perylene diimide. The PEG modification also increased particle biocompatibility and permitted EPR extravasation *in vivo*. After cell internalization, the cell reductive environment (i.e., glutathione) favored the cisplatin release. Besides its cytostatic effect, it activated the nicotinamide adenine dinucleotide phosphate oxidase (NOX) enzyme that transformed the molecular oxygen in O^.−^ furtherly transformed in H_2_O_2_ *via* superoxide dismutase. The hydrogen peroxide could be further transformed in hydroxyl radical through a reaction catalyzed by the ferric ions inducing effective CDT (Yang et al., 2018b) (Figure 2). The synergistic effect of chemotherapy and CDT was confirmed by an improved *in vitro* and *in vivo* tumor cells killing. Qiao et al. (2018) developed a nanotheranostic platform to enhance the temozolomide effect in glioblastoma by reverting the tumor microenvironment immunosuppressive properties through the inhibition of TGF-β expression. The carriers were designed to (1) overcome the blood-brain barrier; (2) target to glioma cells; (3) escape from the endolysosomal compartment; (4) co-deliver temozolomide and the siRNA against TGF-β; (5) be tracked *via* MRI. The system consisted of superparamagnetic iron nanocubes (an excellent MRI contrast agent) encapsulated in poly[(2-acryloyl)ethyl(p-boronic acid benzyl)diethylammonium bromide] polymer loaded with the TGF-β siRNA and coated with zwitterionic lipids coordinating the drug. The particles were additionally modified on their surface with the peptide angiopep-2 targeting low-density lipoprotein receptor-related protein to favor particle translocation through the blood-brain barrier and cancer cell internalization. The system could escape from the endolysosomal compartment through the zwitterionic coating that, in acidic conditions, could acquire a net positive charge destabilizing the membrane of these organelles. Upon interaction with cellular ROS, the benzylboronic acid oxidized reversing the charge of the carriers from positive to negative. This phenomenon induced the release of the therapeutic payloads, and the iron nanoparticles exploited as a contrast agent for MRI.

The application of ROS-responsive theranostics was applied to different conditions. To decrease the generation of H_2_O_2_ in peripheral artery disease that negatively affects the neoangiogenesis process in this condition, Jung et al. generated a new concept of nanotheranostic to detect the diseased tissue through ultrasound and PA imaging (Jung et al., 2019). The system consisted of boronated maltodextrin that, in the presence of tissue H_2_O_2_, released 4-hydroxybenzyl alcohol with proven antioxidant and anti-inflammatory properties (Luo et al., 2017; Tan et al., 2018). Also, upon ROS interaction with the boronate, the system generated CO_2_ bubbles with echogenic properties for ultrasound imaging. The nanoparticles were loaded with indocyanine green (ICG), allowing for multimodal fluorescent and PA imaging. The theranostic properties of these particles were successfully tested *in vitro* and *in vivo* in a model of hindlimb ischemia *via* intramuscular administration. H_2_O_2_ is considered a mild but very common ROS in hepatic ischemia/reperfusion injury, which is a potentially fatal condition for many conditions, including liver transplantation, liver surgical resection, and hemorrhagic shock (Ushitora et al., 2010). Kang et al. (2016) designed polymeric nanotheranostic based on poly(vanillin oxalate) that can serve as a scavenger for H_2_O_2_ while showing anti-inflammatory and antiapoptotic properties. These particles incorporated a prodrug form of vanillin, a compound known for its anti-inflammatory effect, but not extensively investigated in the clinic due to its short half-life. The molecule was loaded in the particles through H_2_O_2_-sensitive peroxalate ester linkers. More importantly, once activated by H_2_O_2_, the peroxalate esters decomposed in CO_2_ bubbles that could be tracked *via* ultrasound imaging in a model of murine hepatic ischemia/reperfusion injury. In this case, the particles were not targeted to exploit their natural tropism toward the liver.

The development of ROS-responsive molecules showed promising results in preclinical testing. Despite their theranostic properties, they can also serve as *in vivo* nanosensors to measure ROS generation. The responsiveness of some material to ROS can be exploited to generate CO_2_ for improving current ultrasound diagnostic methods. Besides, these technologies can be applied to different diseases, since the generation of ROS is a characteristic of various pathological conditions.

## Enzyme Responsive Theranostics

Tissue remodeling and the overexpression of the lytic enzymes that govern this process characterize many pathological conditions. These enzymes can be exploited as triggers since they can favor the degradation of the carriers and the consequent release of the theranostic payloads in the diseased area.

**Figure 3:**
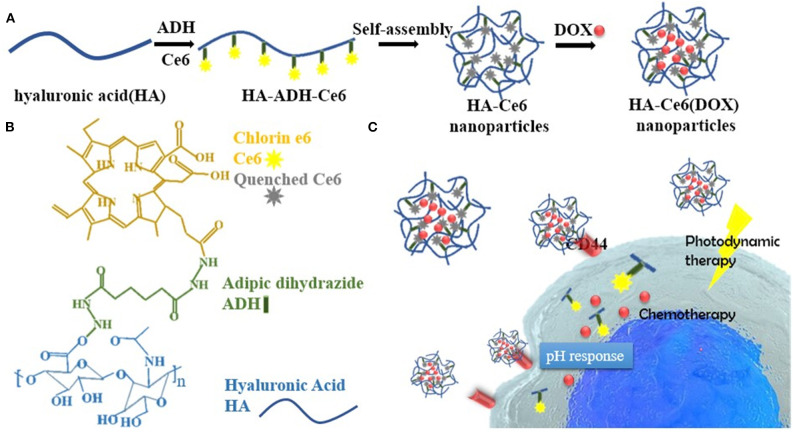
**(A)** Synthetic process of HA-Ce6 (DOX) nanoparticles. Hyaluronic acid (HA) and adipic dihydrazide (ADH) were dissolved in the presence of Ce6-NHS ester and EDCI catalyzing the reaction of particle self-assembly in acidic conditions. Doxorubicin (DOX) loading was performed post-synthesis. **(B)** Illustration of the different components forming the particles. **(C)** the working mechanism of HA-Ce6 (DOX). After intravenous injection, the particles targeted the tumor *via* EPR and were internalized by the cancer cells through the CD44 HA receptor. In tumor microenvironment and cells, the particle could dissolve releasing DOX and Ce6, whose fluorescence was previously inhibited by the presence of a quencher in particle structure. Particle degradation was favored by the acidic conditions acting on the pH-sensitive hydrazine linkers and by the enzyme hyaluronidase degrading the nanoparticle matrix. Reproduced with permission from Ren et al. (2019).

In the case of enzyme-responsive theranostics, are not rare examples of nanoparticles with multiple responsive properties. For instance, Chen et al. developed ferritin nanocages sensitive to pH and matrix metalloproteinase (MMP)-13 activity. The particles, named CMFn@HCQ, were conceived to deliver hydroxychloroquine in the cartilage tissue to ameliorate osteoarthritis conditions (Chen et al., 2019b). In osteoarthritis, the joint microenvironment is characterized by an acidic pH (close to 6) (Li et al., 2017) and MMP-13 overexpression. Ferritin was genetically modified to increase its targeting for collagen II through the addition of a specific peptide in its structure. These carriers were further modified with an MMP-13 cleavable peptide conjugated with the near-infrared (NIR) dye cy5.5 and a quencher to provide the system with enzyme-sensitive diagnostic properties. Because of this chemical modification, only after MMP-13 activity separating the dye and the quencher, the diagnostic signal could be registered. The particles were loaded with the anti-inflammatory hydroxychloroquine, and their structure degraded under acidic conditions inducing the release of the payload. The resulting nanocages had a size of 20 nm, favoring their diffusion in the dense protein matrix of the joint. These carriers demonstrated high therapeutic properties as well as the ability to detect MMP-13 overexpression *in vitro* and *in vivo* when administered *via* intra-articular injections. Ren et al. (2019) engineered a carrier named HA-Ce6 DOX composed of a hyaluronic acid ultrastructure that, despite the ability to offer a natural targeting for the CD44 receptor, overexpressed on cancer cells, also granted the presence of multiple modification sites. The system was conjugated with Ce6 (for PDT and fluorescent detection) and DOX through a pH-sensitive hydrazine bond (Figure 3). The release of the photosensitizer and the chemotherapeutic was further accelerated by the enzyme hyaluronidase, overexpressed in the tumor microenvironment. The enzymatic degradation of the particles was fundamental for the activation of the diagnostic properties of the system since, when encapsulated, Ce6 was affected by the aggregation-caused quenching (ACQ) effect (Li et al., 2020b). CPT-loaded mesoporous silica nanoparticles were functionalized with a cyclo-RGD peptide, and another peptide conjugated with a fluorescent dye and a quencher. Both the peptides were sensitive to the proteolytic action of MMP2. Cyclo-RGD had the function of improving cancer cell targeting and stabilizing the drug in the carriers' pores. When the particles were internalized in the cells, both the peptides were digested inducing the quencher and the dye separation with consequent fluorescent signal detection and drug release (Hu et al., 2016). More investigation will be necessary to test the efficacy of this theranostic platform *in vivo*. Gold nanorods were used to generate a dual stimuli theranostic nanocarrier to provide efficient PTT as well as a detectable diagnostic signal in response to tumor pH and MMPs (Zhao et al., 2017). The gold nanorods were modified with an asymmetric cyanine *via* an MMP sensible linker. This dye could emit near-infrared fluorescence in a pH-dependent manner (Zhao et al., 2015), presenting a reversible “off/on” signal emission as a function of this parameter. Despite its sensitivity for the pH, the gold nanorods represented a significant FÖrster resonance energy transfer (FRET) quencher for the asymmetric cyanine that could emit the fluorescent signal only when in free form. Both the gold nanorods and the asymmetric cyanine allowed for PTT upon irradiation at 808 nm. Finally, drug delivery was achieved through an additional functionalization with glycosyl groups that significantly increased particle biocompatibility, EPR effect, and tumor targetin*g via* GLUT-1 receptor. Liu et al. (2016b) designed a nanotheranostic system based on fluorescent quantum dots embedded in a nanoporous silica matrix with pH-activatable targeting properties and a protease-sensitive drug delivery mechanism. The system was surface modified with a zwitterionic anti-biofouling layer composed of chemical groups with positive and negative charges [COO ^−^ and –HN ^+^ (Me), respectively]. In physiological pH conditions, the particles showed high circulation time, reduced sequestration in the organ of the mononuclear phagocytic system, and reduced protein corona formation due to their neutral surface charge. To this purpose, this strategy was previously demonstrated to be more effective and stable than PEG surface modification (Holmlin et al., 2001; Gui et al., 2013). When exposed to pH below 6.8, like in the tumor microenvironment, they acquired a positive charge favoring their uptake into the tumor cells. The pores of the silica could be loaded with a drug (DOX) and coated with the polymer polycaprolactone that was exploited to stabilize the drug in the particle structure. This polymer was sensitive to the enzyme esterase that is overexpressed and secreted by cancer cells, making tumor microenvironment and cells favorable sites for drug release. Also, it is important to highlight that the enzymatic degradation was further favored at acidic pH, where the particles acquired a positive charge and were more accessible to the enzyme and prone to cell internalization.

The enzyme-responsive theranostic working mechanism usually depends on hydrolytic enzymes favoring carrier or peptide linkers degradation. This phenomenon can also be exploited through coatings used to stabilized the therapeutics or the diagnostic molecules in the particles. The efficient application of enzyme-responsive theranostics strictly depends on the over-expression of some enzymes that can characterize different pathological conditions. Their synthesis relies on the engineering of biological substrates, usually functionalized with other molecules (including targeting modifications). Some of these modifications aim at providing higher biocompatibility and biological interaction, opening new avenues of research in the field of bioinspired nanomedicine.

## Theranostics Responsive to Other Biological Stimuli

**Figure 4:**
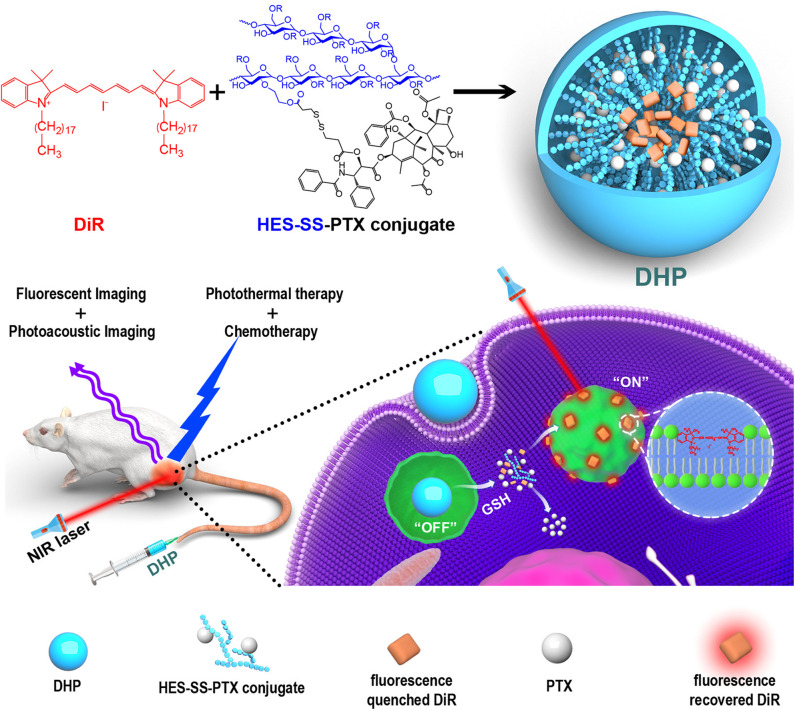
The major components of DHP nanoparticles are the disulfide-bond-linked hydroxyethyl starch paclitaxel conjugate (HES-SS-PTX) and the near-infrared dye cyanine fluorophore DiR. The particles were designed to exert dual imaging (fluorescent and photoacoustic imaging) and dual therapy (photothermal and chemotherapy) properties. The HES shell provided the particles with EPR properties. After intravenous injection, the particles could accumulate in the tumor microenvironment and be internalized inside the cells. In the cell cytoplasm, the disulfide bond degradation occurred due to the cellular GSH that favored the release of PXT (chemotherapy) and DiR. When encapsulated in the particles, DiR was affected by ACQ, but upon its release, it could exert its therapeutic (photothermal therapy) and diagnostic (photoacoustic and fluorescent imaging) properties. Reproduced with permission from Li et al. (2019b).

Glutathione (GSH) is a reactive molecule controlling the cellular redox balance. Cancer cells significantly overexpress this molecule, pointing out its potential role as a targetable trigger for the development of smart nanotheranostics (Liu et al., 2016a). To this purpose, theranostic platforms based on GSH reactivity are usually composed of a targeted particle modified with a disulfide bond coordinating a chemotherapeutic and/or a fluorescent probe (Han et al., 2017). A typical example of GSH-responsive theranostic based on a facile synthesis is represented by nanoparticles generated through hydrophilic polymers conjugated with hydrophobic drugs. The amphiphilic monomers can favor nanoparticle self-assembly, leaving room for further modification with a diagnostic probe. Exploiting these principals, Li et al. (2019b) generated multimodal imaging and therapeutic nanotheranostics named DHP. These carriers consisted of a disulfide-bond-linked hydroxyethyl starch conjugated with the chemotherapeutic paclitaxel (PXT). The addition of dioctadecyl-3,3,3,3-tetramethylindotricarbocyanine iodide (DiR) in the synthesis solution resulted in the entrapment of the dye in the hydrophobic core of the particles through one single step of dialysis. DiR allowed for both fluorescent and PA imaging, and when irradiated with at 808 nm, PTT providing synergistic effects with the chemotherapeutic. When packaged within the nanoparticle structure, the dye was affected by ACQ. However, after particle internalization, the cellular GSH induced the release of the theranostic payloads (Li et al., 2019b) (Figure 4). These properties were proven *in vitro* and *in vivo* in a model of breast cancer. These particles could efficiently accumulate in the tumor *via* EPR due to the high circulation properties offered by the hydroxyethyl starch. Similarly, a GSH-sensitive platform was synthesized through low molecular weight heparin polymerization that, in the presence of cystamine, polymerized with Ce6 in biocompatible carriers (Yang et al., 2018a). In addition, heparin was previously shown to possess antimetastatic and anti-angiogenic properties (Yang et al., 2015; Mei et al., 2017). The system offered sensitivity to a reductive environment since the cystamine linker contained a disulfide bond in its structure. GSH induced particle degradation and Ce6 release activating the theranostic properties of the dye that, when encapsulated, was affected by the ACQ effect. Finally, the system was also loaded with PXT in combination with alpha-tocopherol succinate (Yang et al., 2018a) to provide therapeutic synergism between chemotherapy and PDT. When injected intravenously, the particles accumulated *via* EPR in a model of 4T1 breast cancer. After cell internalization, the particles were degraded by the abundant levels of GSH releasing PXT and Ce6 that in free form was easily detectable through NIR imaging and inducible for PDT. Song et al. (2017) engineered iron oxide nanoparticles to be responsive to ATP, which is highly generated in tumor cells when compared to healthy tissue as well as to a decrease in tissue pH. The system was composed of small clusters of superparamagnetic iron nanoparticles and tannic acid that self-assembled in water-soluble aggregates smaller than 100 nm due to hydrophobic interactions. The particles were modified with ICG and DSPE-PEG *via* non-covalent hydrophobic interactions and hydrogen bonds, respectively. The presence of PEG increased particle circulation and tumor accumulation *via* EPR. The core of iron oxide permitted strong MRI properties while its high affinity for ATP induced particle disassembly in the presence of this molecule (Yu et al., 2013). On the other hand, the ICG permitted particle fluorescence detection after degradation (since the dye was affected by the ACQ effect) as well as PTT applications when irradiated at 808 nm. Particle degradation was further boosted in acidic pH, perhaps due to the protonation of the hydroxyl groups of the tannic acid that lowered the affinity of this molecule for the iron oxide nanoparticle structure. The disassembly of the system was pivotal to activate its imaging properties as well as its renal clearance. The system was successfully tested i*n vitro* and *in vivo* in a subcutaneous tumor model for its theranostic and responsiveness properties obtained through facile synthesis and loading protocols.

Biological responsiveness was also used to ameliorate the conditions of diseases different than cancer. In Alzheimer's disease, the aberrant generation of plaques of soluble β-Amyloid proteins in the central nervous system occurs (Benilova et al., 2012). This phenomenon is favored by the accumulation of metal ions in the tissue like copper, which *via* ROS generation can support protein aggregation (Atwood et al., 2018). Cui et al. designed upconversion NaYF4:Yb/Er/Tm nanocrystals conjugated with 8-hydroxyquinoline-2-carboxylic (HQC) and DSPE-PEG to provide the carriers with diagnostic, therapeutic and biocompatibility properties, respectively (Cui et al., 2016). The particles were designed to (1) detect Cu^2+^ *via* luminescence resonance energy transfer from the particle to the copper, (2) targeting and imaging of the β-Amyloid- Cu^2+^ complexes by registering the upconverted light signal emitted further NIR light irradiation (demonstrated *in vitro* and *ex-vivo*) (3) chelate the copper *via* HCQ. The theranostic properties of this system were successfully proven in a zebrafish model and *ex-vivo*.

These examples demonstrated that any reactive molecules could be exploited as a tool to generate responsive nanoparticles. GSH, in particular, was extensively investigated in literature for this purpose since cancer cells are characterized by a very high content of this molecule. On the other hand, they are also characterized by the high generation of ROS. For this reason, further advances in the field need to take into consideration the efficient alteration of this balance, both when generating GSH- as well as ROS-responsive carriers.

## Conclusions

The fine-tuning of the therapeutic regimens and diagnostic methods represents an emerging need in all the fields of experimental medicine. Current efforts of the scientific community are paving the way to achieve this goal through the generation of materials that are sensitive and can respond to chemical, physical, and biological triggers. Therapeutic and diagnostic properties can reside in the same molecule embedded in the nanocarrier structure that can be activated at the injury site, or they can independently derive from different chemicals loaded into and/or conjugated onto the surface of the nanoparticles. Also, the very same structure of the particles can alternatively provide therapeutic or diagnostic properties. In this work, we presented some examples of pathological stimuli-responsive theranostics with multiple therapeutic and diagnostic properties, sometimes combined with external stimuli-responsive materials (Table 1). The goal of this work was to present the current strategies and materials used for imparting the theranostics with biological-responsiveness.

**Table 1:** Summary table of the different technologies and their theranostic properties.

**Stimulus**	**Nanocarrier**	**Size (nm)**	**Administration/targeting**	**Disease/therapeutic**	**Imaging**	**References**
pH	poly(amidoamine) dendrimers	2.8	N.A./FA vs. FAr	TU/DOX (Ch.T.)	Au NP (CT)	(Zhu et al., 2018)
	graphene oxide/BSA/PheoA NP	182	IV/FA vs. Far	TU/PheoA (PDT)+GO (PTT)	PheoA (FL)	(Battogtokh and Ko, 2016)
	CaCO3 NP	140	IV/EPR	TU/Ce6(PDT)+ Ch.T. (DOX)+Mn (CDT)	Mn (MRI)+ Ce6 (FL)	(Dong et al., 2016)
	MnCO3/FeO/Se NP	100	IV/ EPR	TU/Mn and Se (CDT+limotherapy)	Mn (MRI)	(Xiao et al., 2019)
	magnetic mesoporous nanocubes (MMM)	142	IV//EPR+ magnetic guidance+FA vs. FAr	TU/Vc (CDT)+MMM (HT)	MMM (MRI)	(Li et al., 2020a)
pH+ enzyme	Ferritin nanocages	20	Intrarticular/ pep vs. collII	Osteoarthritis/ hydroxychloroquine (AI)	Cy5.5 (FL)	(Chen et al., 2019a)
	Hyaluronic acid (HA) NP	90	N.A./HA vs CD44	TU/DOX (Ch.T)+ Ce6 (PDT)	Ce6 (FL)	(Ren et al., 2019)
	Mesoporous silica	200	N.A./EPR	TU/DOX (Ch.T.)	Quantum dots (FL)	(Liu et al., 2016b)
pH+ enzyme+NIR	Gold nanorods	50 × 12	IV/glycosyl groups vs. GLUT-1	TU/gold nanorods and asymmetric cyanine (PTT)	Asymmetric cyanine(FL)	(Zhao et al., 2017)
pH + ATP	Fe/tannic acid NP	79	IV/ EPR	TU/ICG (PTT)	Fe (MRI)+ ICG(FL)	(Song et al., 2017)
pH+GSH	BSA/MNO2 NP	160	IV/EPR	TU/CIS (Ch.T)+hafnium (RT)	Mn (MRI)	(Liu et al., 2017)
ROS	superparamagnetic Fe nanocubes	120	IV/angiopep-2 vs LRP1	TU/TMZ (Ch.T)+TGFβ siRNA	Fe (MRI)	(Qiao et al., 2018)
	boronated maltodextrin NP	350	IM/N.A.	Peripheral artery disease/4HBA (Sc,AI)	CO_2_ (US)+ ICG (PA)	(Jung et al., 2019)
	Poly(vanillineoxalate) NP	550	IV/N.A.	Hepatic ischemia/reperfusion injury/ Vanillin (Sc.)	CO_2_ (US)	(Kang et al., 2016)
ROS + GSH	perylene diimide NP	120	IV/EPR	TU/CIS (Ch.T)+/Fe (CDT)	PDI/IR790 (ratiometric PA)	(Yang et al., 2018b)
GSH. + NIR	hydroxyethyl starch NP	160	IV/N.A.	TU/PXY (Ch.T)+DiR (PTT)	DiR (FL+ PA)	(Li et al., 2019b)
	Heparin/cystamine NP	211	IV/EPR	TU/Ce6 (PDT)+PXY (Ch.T.)	Ce6 (FL)	(Yang et al., 2018a)
Cu	upconversion NaYF4:Yb/Er/Tm nanocrystals	27	N.A./Cu	Alzheimer's disease/HCQ (Cu chelation)	luminescence resonance energy transfer	(Cui et al., 2016)
MMP-2	Mesoporous silica	150	N.A./cRGD vs integrin	TU/CPT (Ch.T.)	TAMRA (FL)	(Hu et al., 2016)

*AI, anti inflammatory; Ch.T., Chemotherapy; CDT, Chemodynamic therapy; CPT, Camptothecin; DOX, Doxorubicin; EPR, Enhanced permeability and retention effect; FA, Folic acid; Far, Folic acid receptor; FL, Fluorescence; GO, Graphen Oxide; GSH, Glutathione; HA, Hyaluronic acid; HT, Hyperthermal therapy; IV, intravenous; LRP1, low-density lipoprotein receptor related protein 1; MMP, Matrix metallo proteinases; MRI, Magnetic resonance Imaging; NIR, Nearinfrared light; NP, Nanoparticles; PA, Photoacoustic imaging; PDT, Photodynamic tehrapy; Pep, peptide; PTT, Photheral therapy; PXT, Paclitaxel; ROS, Reactive oxygen species; RT, Radiotherapy; Sc, scavenger; TU, Tumor; US, Ultrasound imaging*.

To our knowledge, no nanotheranostic was FDA approved to date (Anselmo and Mitragotri, 2019). However, clinical trials are currently active to evaluate their efficacy and safety. In this effort, most of the theranostic under clinical trials base their working mechanisms on remotely applied stimuli. In particular, iron (Nardecchia et al., 2019) and gold (Pedrosa et al., 2015) nanoparticles are extensively tested in humans for their multimodal imaging modalities, PDT and PTT properties as well as for their relatively easy synthesis and functionalization. External stimuli-responsive carriers activation strongly depends on the penetration and the precision of the external signal, and most importantly, they can be activated only in the areas that current diagnostic tools detect as disease sites. On the other hand, the responsiveness of theranostics to pathological stimuli theoretically depends solely on their ability to target the environmental conditions of the diseased tissue or cell, with no further interventions of external procedures. In this context, they could reveal and be activated in sick areas that were not detected by other imaging tools. Precise targeting, therefore, represents their main limitation. Due to the complex nature of these technologies, targeting is usually imparted through the pegylation of the carriers and depends on their passive accumulation in the diseased tissue through the EPR effect. However, this kind of targeting could jeopardize their responsiveness. For example, in some cases, the carriers need to be internalized in the cells to perform their theranostic functions while PEG functionalization could affect this process (Moros et al., 2012). The complexity of their synthesis protocols, as well as their low biocompatibility and inherent toxicity, represent other significant limitations in the clinical translation of these technologies (Kunjachan et al., 2015). However, more investigations on the development of theranostic molecules like porphyrins, Ce6, or Mn^2+^ could streamline their synthetic routes while combining the advantages of external- and biological-responsive theranostics. Finally, relying on biological stimuli, they could suffer from unspecific activation in other tissues (i.e., the organs of the mononuclear phagocytic system). For this reason, more research to evaluate their adverse effect, metabolization, and excretion is necessary for future developments.

## Author Contributions

AP and YA: conceptualization. AP and MR writing (original draft preparation). SL review and editing. AZ supervision and funding acquisition.

## Conflict of Interest

The authors declare that the research was conducted in the absence of any commercial or financial relationships that could be construed as a potential conflict of interest.
